# Friendship quality among autistic and non-autistic (pre-) adolescents: Protective or risk factor for mental health?

**DOI:** 10.1177/13623613211073448

**Published:** 2022-01-22

**Authors:** Rachel A.G. O’Connor, Neeltje van den Bedem, Els M.A. Blijd-Hoogewys, Lex Stockmann, Carolien Rieffe

**Affiliations:** 1 Leiden University, The Netherlands; 2 INTER-PSY, The Netherlands; 3 University College London, UK

**Keywords:** anxiety, autism, depression, friendship, mental health

## Abstract

Autistic (pre-) adolescents are often misunderstood by their peers, which can lead to challenges within their friendships. Yet, friendships play an important role in our psychological wellbeing, whereby in the non-autistic population good quality friendships usually protect against mental health difficulties, whereas conflictual friendships increase the risk. The present study investigated positive and negative friendship quality in autistic and non-autistic (pre-) adolescents. Furthermore, the relations between positive friendship quality and negative friendship quality with anxiety and depressive symptoms were examined. Participants were 306 autistic and non-autistic (pre-) adolescents aged 9–16 (*M* = 11.69 years; *SD* = 1.33 years). Our results demonstrated that autistic (pre-) adolescents reported lower positive friendship quality than their non-autistic peers, while no group or gender differences were observed for negative friendship quality than non-autistic boys. For the whole sample, positive friendship quality was associated with fewer depressive symptoms, while the opposite was true for negative friendship quality. For autistic girls only, higher positive friendship quality was related to more symptoms of anxiety. These outcomes seem to emphasize the need for supporting the friendships of autistic young people, perhaps by educating non-autistic young people around how to be supportive friends to their autistic peers.

**Lay abstract:**

Autistic young people are often misunderstood by non-autistic young people, and this can lead to difficulties in their friendships. We know that friendship is very important for our mental health. For non-autistic young people, having good friendships is linked to better mental health and having problems in friendship can cause mental health problems. This study aimed to compare the positive and negative features of friendship that autistic non-autistic young people experience. The study also aimed to understand if having positive or negative friendship features is related to signs of mental health problems (anxiety and depression). 306 young people aged 9–16 took part in this study. These were 86 autistic boys, 18 autistic girls, 91 non-autistic boys and 111 non-autistic girls. The findings of this study showed that autistic young people have less positive friendship features than non-autistic young people. For all young people in the study, having more positive friendship features was related to fewer signs of depression, while having more negative friendship features was related to more signs of depression. Just for autistic girls, having more positive friendship features was related to more signs of anxiety. These findings show that support is needed to help autistic young people have more positive friendships. For example, by teaching non-autistic young people how to be supportive friends to their autistic peers.

## Introduction

Throughout our lifespan, friendships are a crucial source of support and affection, which help us get through difficult situations in life. Friendships offer a sense of social belonging and may be characterized by closeness, love and trust (Hruschka, 2010). Friendship evolves throughout the lifespan, and early adolescence is a time of significant change. It is a time of self-discovery and development of independence from parents, when young people start spending longer days at school and leisure activities with peers (Laursen & Collins, 2012). Friendship becomes more important, but also more complex (De Goede et al., 2009). Young adolescents group together to develop their own culture, where social pressures to conform increase to allow for the smooth functioning of the group (Bronfenbrenner, 1979). In line with this, the quality of young people’s friendships gradually increases throughout childhood and into adolescence (Berndt, 2004; Meeuws, 2016). In particular, the level of support and intimacy within friendships increases significantly in adolescence (Meeuws, 2016).

(Pre-) adolescent friendship requires increased adherence to social expectations, such as inherently understanding another’s wishes and intentions, negotiating personal boundaries and initiation of pro-social actions, many of which are challenging for autistic teens (Bottema-Beutel et al., 2019; O’Connor et al., 2019). In addition, non-autistic people often misunderstand autistic people, leading to negative perceptions of autistic people, perhaps contributing to their social exclusion (Davis & Crompton, 2021; Mitchell et al., 2021). The present study focuses on how autistic (pre-) adolescents may differ from their non-autistic peers in terms of the quality of their friendships. Furthermore, we investigate to what extent friendship quality is related to the mental health of autistic and non-autistic (pre-) adolescents.

### Autism and friendship

Autism is a neurodevelopmental condition comprising social communication differences such as alternative non-verbal communication preferences and a more direct communication style, as well as a preference for sameness, including a preference for routines and focussed, passionate interests (American Psychiatric Association [APA], 2013). Autistic people’s social style and approach are different from non-autistic people, often causing mutual misunderstanding and difficulties with social interactions, which can also bring about problems in friendships (Mendelson et al., 2016; Mitchell et al., 2021). Friendships are complex relationships that involve a variety of both positive and negative interactions and characteristics. Friendships high in positive friendship quality (PFQ) are characterized by a range of important features, including having fun together, feelings of companionship, a reliable alliance, sharing of internal experiences, support in times of trouble and the expression of affection (Kouwenberg et al., 2013). Friendships characterized by high PFQ are considered to be crucial for the adaptive development of young people and are associated with a range of positive outcomes for non-autistic young people, such as increased happiness, fewer behavioural problems and feelings of higher self-worth (Hiatt et al., 2015; Raboteg-Saric & Sakic, 2014; Zucchetti et al., 2015).

Due to their different social preferences and communication style, it is often assumed that autistic people do not have the same desire for friendships, but autistic people tell a very different story (Cresswell et al., 2019). Autistic (pre-) adolescents report how important it is to them to experience friendship and companionship, and to fit in with peers (Calder et al., 2013; Cook et al., 2018; Daniel & Billingsley, 2010; Sedgewick et al., 2016; Sumiya et al., 2018). Unfortunately, they tend to experience lower PFQ than their non-autistic peers. This lower overall PFQ seems to be experienced by (pre-) adolescent boys and girls from the ages of 6 to 16 (Head et al., 2014; Mendelson et al., 2016; Sedgewick et al., 2019). Taking a closer look at specific features associated with PFQ, autistic young people reported they experience less companionship and get less help from their friends than their non-autistic peers. There are also important gender differences, whereby girls (aged 10–18), regardless of diagnosis, have reported higher PFQ than boys (Head et al., 2014; Sedgewick et al., 2019). Specifically, autistic and non-autistic girls have reported higher levels of security and closeness in their friendships (Sedgewick et al., 2019).

Just as friendships have many positive aspects, there can also be issues and problems. Friendships with higher negative friendship quality (NFQ) may be characterized by feelings of jealousy, expressions of dominance, regular conflict, betrayal and competition (Kouwenberg et al., 2013). This side of friendship has not been studied as much in autistic young people, though we do have some understanding of conflict in autistic friendships, which was explored by Sedgewick and colleagues (2019). Autistic young people aged 11–18 took part in this mixed-methods study, which quantitatively examined five components of friendship; companionship, help, security, closeness and conflict (findings in relation to the positive components of friendship are presented above), as well as interviewing the participants around their experiences of friendship and conflict. These autistic young people reported more conflict in their friendships than their non-autistic peers, which has also been observed elsewhere (Whitehouse et al., 2009). Furthermore, the autistic adolescent girls reported more relational conflict, as well as more overall conflict, than autistic boys and non-autistic youth. In fact, autistic boys reported little or no conflict with friends and described that any small disagreements they do have are easily resolved or simply forgotten about. The authors note that this could be due to autistic boys having less intimate friendships when compared to non-autistic boys (Sedgewick et al., 2019). A more comprehensive understanding of NFQ (not just conflict) experienced by autistic young people appears to currently be unexplored in the literature.

### Friendship and mental health difficulties

As children reach middle childhood and continue into adolescence, there is an increase in the prevalence of internalizing disorders, such as anxiety and depression (Wilmshurst, 2005). The occurrence of such internalizing disorders is even more common for autistic young people (Menezes et al., 2018; Schwartzman & Corbett, 2020; Solomon et al., 2012; van Steensel & Heeman, 2017). The literature currently shows that for non-autistic young people, having friends can be protective against these mental health difficulties. In fact, having just one good friend can protect against internalizing symptoms (Bayer et al., 2018; Brendgen et al., 2013). In addition, for children who are generally excluded from the peer group or victimized by peers, friendship can act as a buffer, preventing the development of depressive symptoms (Bukowski et al., 2010; Schmidt & Bagwell, 2007). Not just the presence of a friend but the quality of the friendship is important in protecting against psychological symptoms, whereby friendships with more features associated with PFQ, such as security, companionship and helpfulness, are linked to fewer mental health difficulties (Wood et al., 2017; You & Bellmore, 2012). In contrast, friendships, in which there is conflict, are related to more internalizing symptoms (La Greca & Harrison, 2005; You & Bellmore, 2012).

Could friendships with high PFQ and low NFQ be related to mental health in a similar way for autistic young people? Like non-autistic young people, autistic (pre-) adolescents report that they have friends that are there for them and that they can be themselves around (Cook et al., 2018; Sedgewick et al., 2019). They also mention that their friends provide companionship and enjoy laughing together, features that are typical of non-autistic friendships too (Calder et al., 2013; Sedgewick et al., 2016). On the other hand, friendships do seem to be loaded with extra complications for autistic (pre-) adolescents. They have described anxiety about rejection and keeping up with conversations as well as insecurity about their ability to keep friends (Daniel & Billingsley, 2010; Sedgewick et al., 2019; Sumiya et al., 2018). These worries around friendship may impact their protective power, as they elicit concerns as well as pleasure. Furthermore, autistic girls have reported using more maladaptive strategies when repairing friendships (Sedgewick et al., 2019), as well as experiencing more exhaustion and distress as a result of camouflaging their autistic traits when with their friends (Sumiya et al., 2018), which means friendships may be a lot of work for them, rather than something they can fully enjoy.

The literature reviewed thus far seems to suggest that having friends is important to many autistic children and adolescents (as they are for non-autistic young people), and while many reported developing satisfying friendships, the execution of this can be challenging and often exhausting. A small body of literature has quantitatively investigated the link between friendship quality and internalizing symptoms in autistic young people. Good quality friendships were shown to be related to decreased anxiety and loneliness in autistic adolescents (Chang et al., 2019; Lieb & Bohnert, 2017), while specifically having reciprocity in friendships has been linked to less anxiety and depressive symptoms (Mazurek & Kanne, 2010). Some negative friendship features (i.e. conflict and betrayal) were related to increased depressive symptoms for autistic adolescents in one study, while no relationship was observed between positive features of friendship and depressive symptoms in this study (Whitehouse et al., 2009).

### Present study

The present study aims to expand on the current literature by investigating both PFQ and NFQ and how they relate to anxiety and depressive symptoms for autistic and non-autistic (pre-) adolescents (aged 9–16). In addition, the relation between PFQ and NFQ with anxiety and depression is investigated for each group and gender to identify whether the strength of this relationship differs.

In terms of friendship quality, we expected that autistic individuals would have lower PFQ and higher NFQ than in non-autistic individuals (Head et al., 2014; Sedgewick et al., 2019). We also expected that girls, regardless of diagnosis, would have higher PFQ than boys (Head et al., 2014; Sedgewick et al., 2019), and autistic girls, due to their high reports of conflict (Sedgewick et al., 2019), would have higher NFQ than other sub-groups. We expected that for both autistic and non-autistic young people, PFQ would be negatively related to anxiety and depression and NFQ would be positively related to anxiety and depression (Mazurek & Kanne, 2010; Whitehouse et al., 2009). We also explored whether the strength of these relationships would differ between groups and genders, though no specific directional hypotheses were made in this regard.

## Method

### Participants

The participants of this study were recruited as part of a larger on-going research project on social-emotional development in autistic young people, as well as young people with hearing loss or developmental language disorder (DLD), some results of which have been published elsewhere (e.g. Broekhof et al., 2018; O’Connor et al., 2019). A total of 306 children and adolescents between the ages of 9 and 16 years (*M* = 11.69 years; *SD* = 1.33 years) participated in the present study (see Table 1). Participants with an IQ below 70 were excluded from the current study (two autistic participants and four non-autistic participants were excluded on this basis). Of this sample, 104 autistic (pre-) adolescents (86 males and 18 females) were recruited from the centre of autism, special education schools and other support organizations. All individuals in this group were diagnosed by trained psychologists, independently of this study, according to the *Diagnostic and Statistical Manual of Mental Disorders (4th ed., text rev.; DSM-IV-TR*; APA, 2000; this was the current version of the *DSM* at time of diagnosis) and based on the Autism Diagnostic Interview – Revised (Lord et al., 1994). The comparison group consisted of 202 non-autistic (pre-) adolescents (91 males and 111 females). Participants in this group were recruited from mainstream primary and secondary schools and were not part of other groups of interest to the larger research project (i.e. they did not have hearing loss or DLD).

**Table 1.** Participant characteristics by sub-group: autistic and non-autistic girls and boys.

	Girls		Boys	
	Autistic	Non-autistic	Autistic	Non-autistic
N	18	111	86	91
Age: *M* (*SD*)	11.37 (1.15)	11.57 (1.27)	11.89 (1.46)	11.71 (1.31)
PIQ: *M* (*SD*)	99.24 (17.67)	107.76 (17.91)	109.30 (19.28)	105 (18.99)
SRS: Total (*SD*)	88.4 (17.55)	28.6 (13.65)	89.7 (28.6)	32.5 (20.15)

PIQ: performance IQ; SRS: social responsiveness scale.

Independent t-tests revealed that autistic girls scored lower on the performance IQ indication score (PIQ) than autistic boys, *t*(24.91) = 2.10, *p* = 0.046, *d* = 0.544. No other differences were found in terms of PIQ (see Table 1). Autistic boys and girls had higher scores on the social responsiveness scale (SRS) than non-autistic boys and girls (*t*(122.36) = –14.00, *p* < 0.001, *d* = 2.31; *t*(97) = –15.23, *p* *<* 0.001, *d* = 3.80, respectively), indicating higher levels of autistic traits.

### Materials

Friendship quality was assessed using the best friend index (BFI) for children and adolescents (Kouwenberg et al., 2013). The BFI is a self-report instrument that measures both PFQ and NFQ within the context of actual friendships. The questionnaire begins by asking participants whether they have a best friend and what their best friend’s name is. Participants are encouraged to keep this particular friend in mind when completing the questionnaire. The BFI consists of 11 items assessing positive friendship features (companionship, reliable alliance, disclosure, support and affection/ admiration; e.g. *I share with my best friend*) and nine items assessing negative friendship features (jealousy, dominance, conflict, betrayal and competition; e.g. *my friend tries to boss me around*). Participants are asked to rate these statements about their friendship on a scale from 1 to 3 (1 = never true, 2 = sometimes true and 3 = often true). Mean PFQ and NFQ scores were calculated for each participant, whereby higher scores indicated more positive and negative friendship features, respectively. The BFI scales have previously shown good external validity (Kouwenberg et al., 2013). Both the PFQ and NFQ scales have acceptable reliability in the present autistic/ non-autistic sample (*α* = 0.74/0.64 for PFQ and *α* = 0.75/0.61 for NFQ).

Symptoms of depression were measured using an adapted Dutch version of the Child Depression Inventory (Kovacs, 1985), which is a 27-item self-report questionnaire. The items are related to specific symptoms of depression (e.g. *I feel like crying; I get annoyed*), where responses are on a three-point scale (1 = not true, 2 = a bit true and 3 = most of the time true). The item relating to suicidal ideation was removed from the measure, so as not to cause upset to the young people. Thus, the analysis included 26 items. Mean depression scores were calculated for each participant, whereby higher scores indicated higher levels of depression symptomology. The scale was found to have an acceptable internal reliability in the current autistic and non-autistic samples (*α* = 0.77 and *α* = 0.75, respectively).

Symptoms of anxiety were measured using the generalized anxiety disorder sub-scale of the child symptom inventory (CSI; Gadow & Sprafkin, 1994), which was completed by parents. This subscale consists of seven items related to their child’s symptoms of generalized anxiety over the previous 6 months (e.g. *is very tense or cannot relax*), which parents rate on a scale from 1 (=never) to 4 (=very often). Mean anxiety scores were calculated for each participant, whereby higher scores indicated more feelings of anxiety. The internal reliability of this scale in the present study was acceptable for both autistic and non-autistic participants (*α* = 0.79, *α* = 0.79, respectively).

PIQ for autistic participants was typically measured as part of their autism assessment using either the performance WISC, SON-r, Wechsler nonverbal score, Dutch intelligence for education symbolic scale or the Dutch differentiation test. For autistic or non-autistic participants with no PIQ score available, an IQ indication score was calculated using two subtests of the WISC (Kort et al., 2002; Wechsler, 1991): block patterns and picture arrangement. All raw scores were converted into age-equivalent scores based on Dutch standard scores.

The SRS (Constantino & Gruber, 2005) was completed by parents, to measure autistic traits. The scale consists of 65 items with responses on a 4-point scale (0 = never true; 3 = almost always true), where higher scores indicate more autistic traits. The SRS measures four areas of social functioning, based on parent observations (social cognition, social motivation, social communication and social awareness), as well as autistic mannerisms.

### Procedure

Permission for this study was granted by the ethics committee Leiden University, Department of Psychology, and written informed parental consent was gained from all parents of the participants beforehand. The self-report questionnaires were administered to the participants in a quiet room at the children’s school or home, by a trained researcher following a detailed protocol. Test sessions lasted approximately 1 h. Parents were asked to complete questionnaires online or on paper. The measures described in the present study are part of a larger research project, which includes additional measures that are not reported here.

### Statistical analysis

#### Missing data

For the friendship variables, 26 participants did not complete the BFI. For 18 of these participants (2 non-autistic boys, 11 non-autistic girls, 4 autistic boys and 1 autistic girl), this was because they did not have a best friend. For the remainder of the non-completed BFI (8), no reason was given. As friendship quality is the primary variable of interest in the present study, cases where the BFI was missing were removed from the data set and not included in any descriptive data or analysis described in this article.

PIQ scores were not obtained for 32 participants (24 non-autistic participants and 8 autistic participants). Parent questionnaires were missing in several cases due to non-completion, leading to the anxiety variable being missing (14 non-autistic boys, 25 non-autistic girls, 15 autistic boys and 2 autistic girls). A Little’s (1988) test of missing completely at random (MCAR) was conducted, which revealed that missing values were missing completely at random, χ^2^ (8053, *N* = 360) = 4556.71, *p* = 1.00. To reduce potential bias and maintain statistical power, multiple imputation was employed to reconstruct missing values for the PIQ and anxiety variables. SPSS was used to conduct the fully conditional specification method of multiple imputation, whereby 10 imputations were preformed, and pooled results were used for analysis. There were no differences between outcomes with the original and imputed data.

#### Assumptions

Data within the PFQ, NFQ and depression variables violated the assumption of normality. Furthermore, the assumption of equality of covariance matrices was not met for PFQ or NFQ. Due to this, we also performed non-parametric tests, which are more robust in non-normally distributed data.

#### Analysis

Due to the data being non-normally distributed, a robust version of a 2 × 2 between-subjects ANOVA (Wilcox, 2012) was used to compare groups (autistic/non-autistic) and genders (male/female) with regard to PFQ, NFQ, anxiety and depression. The tests applied 20% trimmed means, which has been shown to achieve a similar level of power as the mean from a normal distribution, and provided a smaller standard error when there are outliers (Mair & Wilcox, 2020). *R* (R Core Team, 2020) was used to perform these tests with the *t2way* function in the *WRS2* package (Mair et al., 2017). Robust ANOVA tests from this function produced a test statistic *Q*, which is approximately F-distributed. The size of each effect was estimated using the explanatory measure of effect size ξ proposed by Wilcox and Tian (2011). This measure does not assume equal variances, and is based on the concept of explanatory power from regression analyses. Values of ξ = 0.10, 0.30 and 0.50 are considered small, medium and large effect sizes, respectively.

Second, a series of Spearman’s correlations were conducted to assess the relation between PFQ and NFQ, with anxiety and depression. Correlations were conducted separately for group (autistic/non-autistic) and gender (male/female) as well as for the whole sample (see Table 3). In addition, the correlations were controlled for age and level of autistic traits (SRS), which did not change the pattern of results and were therefore not reported. In addition, 95% Confidence intervals (CIs) were calculated for each correlation coefficient (also reported in Table 3). Finally, to evaluate whether the strength of correlations varied significantly between sub-groups, we observed whether the CIs of the correlation coefficients overlapped with each other, where overlapping CIs indicate no significant difference and non-overlapping CIs indicate a significant difference. This conservative method of comparison using 95% CIs was used to account for the unequal standard errors between the groups.

#### Community involvement

There is no community involvement in this study.

## Results

### Group and gender differences in friendship quality

See Table 2 for an overview of PFQ, NFQ, depression and anxiety scores for each sub-group. The 2 × 2 robust ANOVA for PFQ showed a main effect for group, Q = 14.95, *p* = 0.001, ξ = 0.51, with autistic young people reporting lower PFQ than non-autistic young people. In addition, there was a main effect for gender, Q = 6.22, *p* = 0.020 ξ = 0.50, whereby girls reported higher PFQ than boys. There was no group × gender interaction, Q = 0.54, *p* = 0.469, ξ = 0.30. The 2 × 2 ANOVA for NFQ showed no group, Q = 3.91, *p* = –0.063, ξ = 0.30, gender, Q = 0.67, *p* = 0.424, ξ = 0.13, or interaction effects, Q = 0.73, *p* = 0.406, ξ = 0.11.

**Table 2.** Mean scores (standard deviation) for PFQ, NFQ, depression and anxiety by groups and sub-groups.

	Min –max	Girls			Boys			Total		
		Autistic	Non-autistic	Total	Autistic	Non-autistic	Total	Autistic	Non-autistic	Total
PFQ	1–3	2.52 (0.32)	2.72 (0.20)	2.69 (0.23)	2.42 (0.32)	2.59 (0.24)	2.51 (0.29)	2.44 (0.32)	2.66 (0.23)	2.59 (0.28)
NFQ	1–3	1.25 (0.26)	1.20 (0.21)	1.21 (0.22)	1.35 (0.32)	1.19 (0.18)	1.27 (0.27)	1.33 (0.31)	1.20 (0.20)	1.24 (0.25)
Depression	1–3	1.44 (0.21)	1.30 (0.20)	1.32 (0.20)	1.41 (0.21)	1.33 (0.18)	1.37 (0.20)	1.41 (0.21)	1.32 (0.19)	1.35 (0.20)|
Anxiety	1–4	2.05 (0.44)	1.43 (0.39)	1.55 (0.45)	2.01 (0.60)	1.41 (0.39)	1.73 (0.57)	2.02 (0.57)	1.42 (0.38)	1.65 (0.53)

NFQ: negative friendship quality; PFQ: positive friendship quality.

The 2 × 2 robust ANOVA for depression showed a main effect for group, Q = 12.37, *p* = 0.002, ξ = 0.32, with autistic young people reporting more depressive symptoms than non-autistic young people. There was no main effect for gender, Q = 0.02, *p* = 0.903, ξ = 0.20, nor an interaction effect, Q = 2.55, *p* = 1.22, ξ = 0.37. The 2 × 2 ANOVA for anxiety showed a main effect for group, Q = 90.29, *p* = 0.001, ξ = 0.77, whereby autistic young people reported more anxiety symptoms than non-autistic young people. There was no main effect for gender, Q = 1.10, *p* = 0.303, ξ = 0.22, nor an interaction effect, Q = 0.06, *p* = 0.812, ξ = 0.65.

Please note that similar results in relation to anxiety and depression in sub-groups of this sample have also been published elsewhere (Bos et al., 2018).

### Correlations between variables

**Figure 1.**
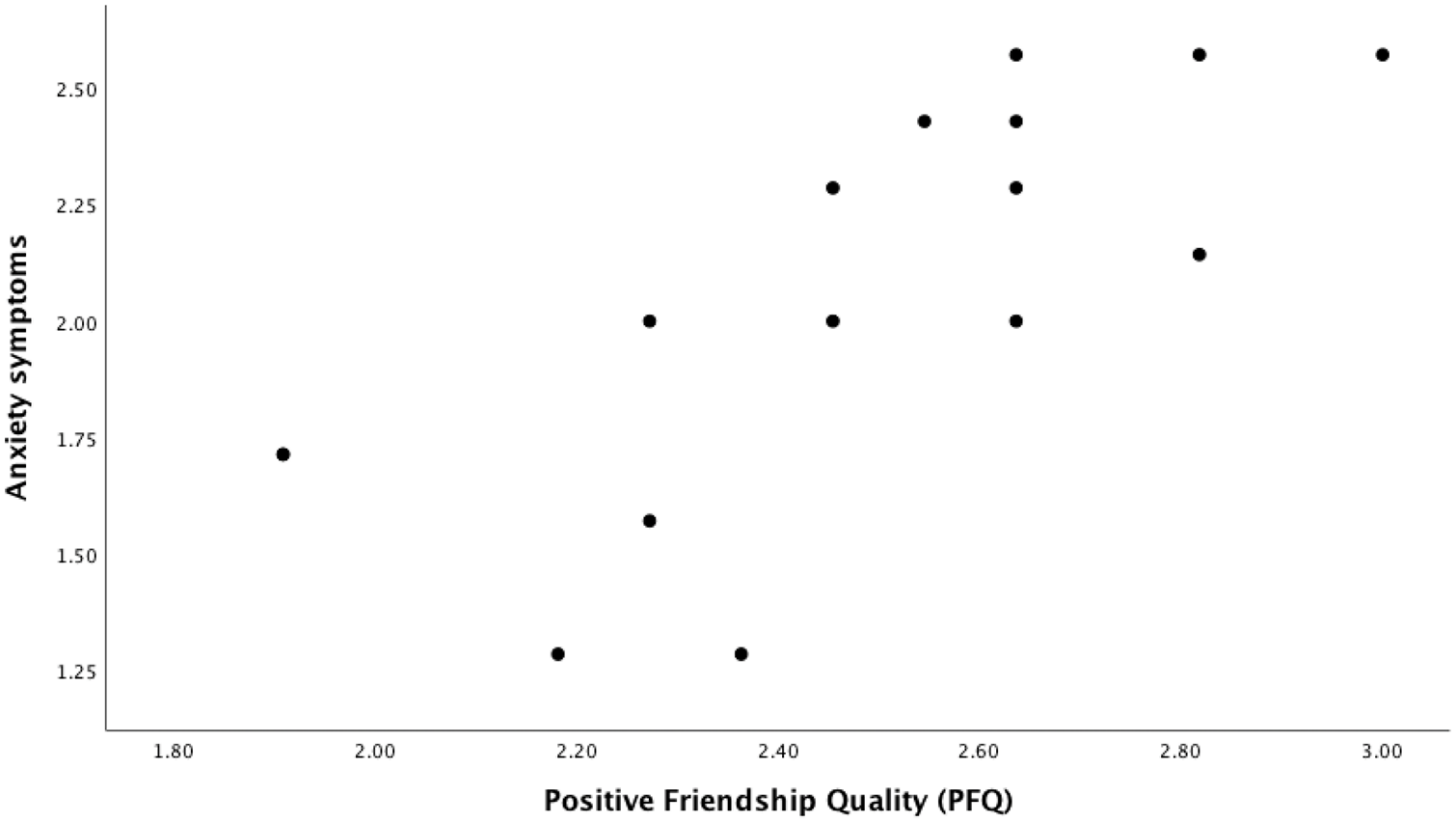
Correlation between PFQ and anxiety symptoms for autistic girls.

Correlations, as reported below, are presented in Table 3. For the whole sample, anxiety was negatively correlated with PFQ, *r_s_* = –0.15, *p* = 0.012, while there was no significant correlation between anxiety and NFQ. Within the sub-groups, there was no significant correlation between anxiety and PFQ for non-autistic (pre-) adolescents or autistic boys, while there was a significant positive correlation for autistic girls, *r_s_* = 0.73, *p* = 0.007. Our data are most consistent with an *r_s_* value for the general population of autistic girls between 0.40 and 0.89 (see Figure 1). This CI does not overlap with CIs for the other sub-groups, indicating a significant difference in the strength of the correlation between anxiety and PFQ for autistic girls, compared to other sub-groups (see Figure 2 in supplementary material showing correlations for all sub-groups). There was no significant correlation between anxiety and NFQ for any sub-groups.

**Table 3.** Correlations: PFQ and NFQ with anxiety and depression.

			Anxiety *r_s_* (95% CI)	Depression *r_s_* (95% CI)
Whole sample	PFQ		−0.15* (−0.25, −0.04)	−0.31** (−0.41, −0.21)
	NFQ		0.06 (−0.06, 0.17)	0.23** (0.13, 0.34)
Girls	PFQ	Autistic	0.73** (0.40, 0.89)	−0.21 (−0.62, 0.28)
		Non-autistic	−0.06 (−0.24, −0.13)	−0.24* (−0.41, −0.06)
	NFQ	Autistic	−0.40 (−0.73, 0.08)	0.48* (0.02, 0.77)
		Non-autistic	0.05 (–0.14, 0.23)	0.28** (0.10, 0.44)
Boys	PFQ	Autistic	0.19 (−0.02, 0.39)	−0.37** (−0.54, −0.17)
		Non-autistic	−0.18 (−0.37, 0.03)	−0.14 (−0.34, 0.07)
	NFQ	Autistic	−0.07 (−0.28, 0.14)	0.17 (−0.04, 0.37)
		Non-autistic	−0.07 (−0.27, 0.14)	0.07 (−0.14, 0.27)

NFQ: negative friendship quality; PFQ: positive friendship quality.

* Statistical significance at *p* < 0.05.

** Statistical significance at *p* < 0.01.

For the whole sample, depression was negatively correlated with PFQ, *r_s_* = −0.31, *p* < 0.001 and positively correlated with NFQ, *r_s_* = 0.23, *p* < 0.001. Within the sub-groups, correlation coefficient CIs for all sub-groups overlap for both PFQ and NFQ, indicating no significant difference in the strength of the correlations between sub-groups.

## Discussion

Developing good quality friendships during (pre-) adolescence is beneficial for overall development and functioning – this is well known in relation to the non-autistic population (Hiatt et al., 2015). The present study aimed to understand whether autistic young people experience similar friendship quality, in terms of both PFQ and NFQ, compared to their non-autistic peers. Another aim of the study was to understand whether PFQ and NFQ can protect autistic young people’s mental health to the same extent as they do for non-autistic young people. In line with previous literature (Mendelson et al., 2016; Sedgewick et al., 2019), the results of the present study indicate that autistic (pre-) adolescents experience lower PFQ than their non-autistic peers. Furthermore, girls report higher PFQ than boys, which is in line with previous studies of both autistic and non-autistic young people (Head et al., 2014; Sedgewick et al., 2019). Autistic and non-autistic boys and girls were found to have similar levels of NFQ, which is inconsistent with previous studies, which have indicated that autistic young people’s friendships have higher levels of conflict, with autistic girls reporting more conflict in their friendships than any other sub-group (Sedgewick et al., 2019; Whitehouse et al., 2009). Finally, depressive symptoms were related to higher PFQ and lower NFQ, while anxiety symptoms were unrelated to friendship quality for all except autistic girls, whose anxiety symptoms were related to higher PFQ.

Autistic young people report a strong desire for friendships (Calder et al., 2013; Cook et al., 2018; Daniel & Billingsley, 2010; Sedgewick et al., 2016; Sumiya et al., 2018) and indeed the large majority of autistic participants recruited in the present study had a best friend, that is, all but one autistic girl and four autistic boys. For comparison, 11 non-autistic girls and 2 non-autistic boys reported not having a have best friend. However, our findings indicate that autistic boys and girls experience less of the positive features of friendship than their non-autistic peers. This may suggest that autistic young people receive less companionship and support from their best friends, as well as having fewer experiences of mutual affection and intimate conversations. Contrary to the predictions of the present study, autistic young people did not report more negative aspects in their friendships.

As expected, good quality friendships were related to fewer depressive symptoms for autistic and non-autistic boys and girls. Similarly, negative friendship features were associated with more depressive symptoms. This is in line with previous studies (Mazurek & Kanne, 2010; Whitehouse et al., 2009) and lends support to the hypothesis that both positive and negative features of friendship are linked to depressive symptoms. The findings are also comparable with studies of non-autistic participants (Wood et al., 2017; You & Bellmore, 2012). For non-autistic young people, this relationship is causational, whereby having a reciprocal best friend is protective against developing depressive symptoms (Bukowski et al., 2010). A similar phenomenon may be represented within the present study, for both non-autistic and autistic young people, but this cannot be confirmed here. This would certainly be a positive conclusion and may suggest that friendships are a helpful and supportive resource for autistic (pre-) adolescents, as they are for their non-autistic peers. On the other hand, this result may indicate that young people with more depressive symptoms struggle to develop or maintain good quality friendships, a scenario which has also been observed in non-autistic adolescents (Schwartz-Mette et al., 2021). If this is the case, this could be a particular worry for autistic young people, who experience more depressive symptoms than non-autistic young people, which was found in the present study, among many others (Gadow et al., 2012; Solomon et al., 2012), thus having a greater impact on friendships. Future research examining causational links would be necessary to tease this out.

Somewhat unexpectedly, for autistic girls, higher PFQ was related to *higher* anxiety, though no relations between anxiety and friendship quality were observed for non-autistic (pre-) adolescents or autistic boys. Due to the small sample of autistic girls, it is impossible to accurately estimate the strength of the relation between PFQ and anxiety (or if there is a true relation at all); our findings show it could be a relatively weak link or a very significant one. Our findings do suggest that the relationship identified between PFQ and anxiety for the autistic girls is not shared with autistic boys or non-autistic (pre-) adolescents. Due to the ambiguous nature of this finding, we can only propose some potential explanations for our results that may stimulate further investigation, without making any definite assumptions. Possibly, maintaining good quality friendships with peers is more stressful for autistic girls, leading to symptoms of anxiety. Young autistic girls have reported regularly falling out with and reforming their peer groups, feelings of insecurity in friendships and worries about maintaining friendships (Cook et al., 2018; Sedgewick et al., 2019; Sumiya et al., 2018). In addition, society may put pressure on autistic girls to camouflage their autistic traits, causing them to work hard to behave in a ‘neurotypical’ manner, which we know from self-reports is a stressful process for autistic people (Hull et al., 2017; Sumiya et al., 2018) and can be related to poorer mental health in autistic adolescents and adults (Bernardin et al., 2021; Cage & Troxell-Whitman, 2019; Cassidy et al., 2018).

### Study strengths and limitations

Prior to the current study, there was scant research examining the link between friendship quality and internalizing symptomology in autistic (pre-) adolescents. It should be noted, however, that the generalizability of this study may be compromised by the small sample of autistic girls (18). Moreover, the sample of autistic girls in the present study may comprise a specific sub-type: those presenting with more ‘typical’ autistic traits or lower IQ, which allowed them to be diagnosed at an early age. Therefore, our findings may only apply to this sub-group of autistic girls and further studies addressing the topics of this study are necessary to confirm or contradict our findings. Furthermore, the present study employed a self-report measure for depressive symptoms and a parent-report measure for anxiety symptoms. This may have produced inconsistent findings if we were to compare depressive and anxiety symptoms, as it is often found that parents’ reports do not reliably match self-reports of young people (Achenbach et al., 1987; though this may not be the case for autistic girls and their parents (Pisula et al., 2017)). Finally, the rather low level of internal consistency within the two BFI scales in the non-autistic group in this study may have impacted our results. The BFI did show good internal consistency in the article that validated this questionnaire (Kouwenberg et al., 2013), so it was possibly the smaller sample size in the present study that contributed to the lower internal consistencies (Field, 2013). Thus, replicating the present study with larger samples may be useful.

### Future research

While we endeavour below to make appropriate practical recommendations, such recommendations may be premature considering the cross-sectional nature of the present study and the limited research currently available. Thus, future research could further investigate the link between anxiety and friendship quality, perhaps through a combination of qualitative studies to understand autistic girls’ and boys’ lived experiences and longitudinal quantitative research to understand any potential causational link. In particular, when considering the results of the present study in the context of the available literature, worthwhile factors to consider may include camouflaging, social stigma and self-stigma. In addition, recent research indicates that autistic people find it easier to communicate with other autistic people, compared to communicating with non-autistic people (Crompton et al., 2020). Thus, it would be useful to explore whether autistic young people have better quality friendships with other autistic people.

### Implications

Our findings suggest that first and foremost, most autistic (pre-) adolescent boys and girls report having a best friend and experience many positive aspects within these friendships. In addition, they report similar levels of negative friendship features as their non-autistic counterparts. However, when compared to their non-autistic peers, their friendships have less positive features. Meanwhile, our results also indicate that, as for non-autistic young people, good quality friendships play a protective role against depressive symptoms for autistic (pre-) adolescents. Alternatively, depressive symptoms may make it more difficult to develop good friendships. Perhaps either way, our findings highlight the importance of supporting the development and maintenance of friendships for autistic young people.

Specific support around friendships is not widely available, as highlighted by a recent systematic review showing the need for supports to move away from teaching (neurotypical) ‘social skills’ and towards specifically supporting autistic young people to develop and maintain fulfilling friendships (Rodda & Estes, 2018). When addressing this issue, we must remember that communication and friendship are two-way streets (Davis & Crompton, 2021); so, non-autistic young people may require education and advice around being supportive friends to their autistic peers. Considering the link between friendship and anxiety in autistic girls observed in the present study, the psychological wellbeing of young autistic people must be at the centre of any support offered around friendships. We must not assume that the problem lies within the autistic people, but rather consider that the non-autistic environment, which is often the dominant majority, may need to adapt and be more focused on inclusiveness and accepting of diversity, thus preventing unnecessary adverse outcomes for autistic people. For example, providing education about autism for schools and communities and promoting anti-stigma campaigns and interventions (e.g. Ranson & Byrne, 2014). Furthermore, it should be highlighted to parents and educators that while many autistic girls may appear to be coping well and making friends, they may in fact be suffering in silence and internalizing their stress and worry.

## Supplemental Material

sj-doc-1-rop-10.1177_13623613211073448 – Supplemental material for Friendship quality among autistic and non-autistic (pre-) adolescents: Protective or risk factor for mental health? Click here for additional data file. Supplemental material, sj-doc-1-rop-10.1177_13623613211073448 for Friendship quality among autistic and non-autistic (pre-) adolescents: Protective or risk factor for mental health? by Rachel A.G. O’Connor, Neeltje van den Bedem, Els M.A. Blijd-Hoogewys, Lex Stockmann and Carolien Rieffe in Autism
