# Supplementary figures and images for: Friendship quality among autistic and non-autistic (pre-) adolescents: Protective or risk factor for mental health?
Source: Autism. 2022 Jan 22;26(8):2041–51. doi: 10.1177/13623613211073448 (PMC9597130; doi:10.1177/13623613211073448)

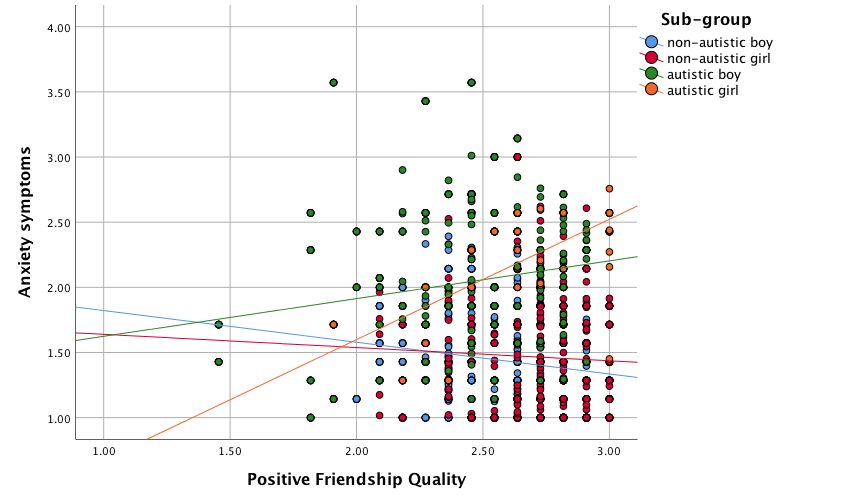


Figure 2. Correlation between PFQ and anxiety (all sub-groups)

Supplement: sj-doc-1-rop-10.1177_13623613211073448 – Supplemental material for Friendship quality among autistic and non-autistic (pre-) adolescents: Protective or risk factor for mental health? [file sj-doc-1-rop-10.1177_13623613211073448.doc]
